# Parallel multigrid method for solving inverse problems

**DOI:** 10.1016/j.mex.2022.101887

**Published:** 2022-11-01

**Authors:** H.K. Al-Mahdawi, A. I Sidikova, Hussein Alkattan, Mostafa Abotaleb, Ammar Kadi, El-Sayed M El-kenawy

**Affiliations:** a Electronic Computer Centre, University of Diyala, Diyala ,32001, Iraq; b Department of System Programming, South Ural State University, Chelyabinsk 454080, Russia; c Department of Food and Biotechnology, South Ural State University, 454080 Chelyabinsk, Russia; d Department of Communications and Electronics, Delta Higher Institute of Engineering and Technology, Mansoura, 35111, Egypt

**Keywords:** Multigrid, Inverse problem, Iteration method, Parallel

## Abstract

We considered in this work the linear operator equation and used the Landweber iterative method as an iterative solver. After that, we used the multigrid method as an optimization method for obtaining an approximation solution with a highly accurate and fast process. A new parallel algorithm for the multigrid process has been developed. The proposed algorithm is based on a V-cycle mixed with the two-grid method. This modification of the V-cycle provides for parallel computing for each level. A coarse grid operator with a residual right-hand side vector for each coarse grid is provided. This parallel algorithm is used to accelerate and enhance computation for the solution of the iteration method in solving the inverse ill-posed problems. The necessary cost-time computation for all stages and processes for the parallel V-cycle algorithm has been done. A numerical experiment on solving the IVP (initial value problem) for the heat equation showed that the new parallel algorithm is much more efficient than the sequential method.

• The study of iteration algorithms and mathematical experiments reveals a slow rate of convergence.
• The Multigrid method is often used to speed up the rate of convergence of iterative methods, which is an effective method of solving large systems of linear algebra equations.
• The approximation solution for the linear algebra equations was found by using the parallel method with the multigrid method.

## Graphical abstract

**Figure:**
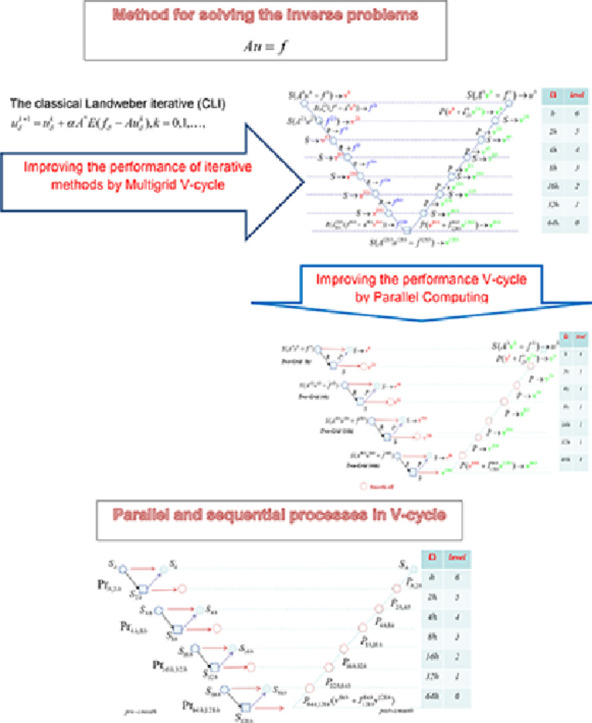

**Specifications table**

**Table:** 

**Subject area**	Computer Science
**More specific subject area**	*Describe narrower subject area*
**Name of your method**	Parallel Multigrid Method
**Name and reference of original method**	• K. Stüben and U. Trottenberg, “Multigrid methods: Fundamental algorithms, model problem analysis and applications,” pp. 1–176, 1982, doi: 10.1007/bfb0069928. • J. H. Bramble, *Multigrid methods*. Routledge, 2019. • P. Wesseling, “An introduction to multigrid methods,” *Math. Comput.*, vol. 57, no. 195, p. 441, 1991, doi: 10.2307/2938685.
**Resource availability**	N.A.

## Introduction

We defined a parallel multigrid algorithm for solving IP (Inverse Problems). In this article, we consider the initial value problem of the one-dimensional (1-D) heat equation as a model of an ill-posed inverse problem. In the early twentieth century, Hadamard [1] labeled situations as well-posed problems, stating that a problem was well-posed when it fulfilled the following points:

1 A solution exists.
2 uniqueness. This solution is unique.
3 Stability (the given data is continuously dependent on the solution).

If at least one of the above points or conditions is not fulfilled in the problem, the problem is considered as an ill-posed problem. The violations of 1 and 2 can often be improved with a small re-formulation of the problem. Violations of stability are much harder to remedy because they imply that a small disturbance in the data leads to a large disturbance in the estimated solution [2], [3], [4], [5]. Various methods and algorithms for solving IP “inverse problems” have been explained and used in [6], [7], [8], [9], [10], [11], [12], [13], [14], [15], [16]. The success of these methods and algorithms is largely based on understanding and analyzing the mathematical problems related to the declarations of the properties of these IP “inverse problems” and identifying specific difficulties in solving them [17], [18], [19], [20], [21], [22].

One of the many known methods of solving a linear operator equation is the iteration method. This method has several shortcomings, including initial guessing, complexity, and convergence. The discretization process for the integral equation primes to a huge, sparse, and high-dimensional system of linear equations or linear operator equation. Inappropriately, theoretic convergence studies and mathematical experimentation show that the convergence rate is slow for these iterative algorithms, leading to an amplified cost of iteration. The direct iteration method suffers from some restricting boundaries. Multigrid methods advanced from efforts to overcome these limitations. Multigrid settings are largely successful when used in conjunction with relaxation or iteration methods, and they lead to fast and direct points to estimated solutions to solve the IP “inverse problems”.

Multigrid methods [23], [24], [25] are frequently used to accelerate the convergence rate of iterative methods [26]. The main idea of this work is to apply parallel computing to the classical multigrid method and consider the cost of parallel and equational computing. All these steps have been applied through the following sections. In Section 2 we defined the inverse problem for linear operator equations with the necessary mathematical notions. In section 3, the classical multigrid method has been defined with some classical algorithms. The new parallel multigrid method has been defined with the cost of computing. In the last section, the numerical examples have been applied and many results have been obtained.

## Problem statement

Let's define the operator A∈H and the vectors u,f∈Hthen let's consider the linear system of equations.

(1) Au=f,

A is linear a compact operator A:H→H. We are interested in cases in which the operator A is obtained from the discretization of an ill-posedness, such as the integral equation of first kind. Express the ill-posed problem of finding an approximate solution to the Eq. (1) as shown in the following.

Suppose that $f=f_{0}$ there exists an real solution $u_{0}$ of problem (1) and this solution is related to the set $M_{r}$which is defined as the class of correctness of (1),

(2) $$M_{r}=\left\{u_{0}:u_{0}\in H,∥u_{0}∥\leq r\right\},$$

but the vector$f_{0}$ is unknown. Instead $f_{\delta }$ and error level or measurement noise δ are given such that

(3) $$∥f_{\delta }-f_{0}∥\leq \delta .$$

Using the given information $M_{r},f_{\delta }$, and δ, we go to find the estimated solution $u_{\delta }$ of (1) and compute the deviation from the real solution $∥u_{\delta }-u_{0}∥$.

The iterative regularization method Landweber [27] used to find the estimated solution $u_{\delta }$defined by next formula

(4) $$u_{\delta }^{k+1}=u_{\delta }^{k}+\alpha A^{*}E\left(f_{\delta }-Au_{\delta }^{k}\right),k=0,1,\ldots ,$$

where α is defined as the regularization parameter and E is the identity operator.

The classical Landweber iterative method (CLI) can be defined by the following algorithm.

*Algorithm 2 CLI (iteration number)*

1 $u^{0}=\bar{0},zeros\;vector\;.$
2 $u^{k}=u^{0}$
3 fork=1,2,3,…iterationnumber
  a $u^{k}=u^{k}+\alpha A^{T}\left(f_{\delta }-Au^{k}\right)$
  b $variational={∥Au^{k}-f_{\delta }∥}^{2}$
4 Endforloop

There are two significant measures of $u_{\delta }$ as an approximation of $u_{0}$ the error and residual. An iterative method for the solution of (1) can be applied directly to the Eq. (1) or to an equation of the error. It is called residual equation [28]. Let $u_{\delta }$ be an approximation of $u_{0}$, then the error $e=u_{0}-u_{\delta }.$ satisfies the equation

(5) $$Ae=f_{\delta }-Au_{\delta }=r,$$

where *r* is the residual, e.g. the volume by which the estimate $u_{\delta }$ fails to satisfy the original problem (1).

## Multigrid method

*Multigrid method* (MG) may be observed as a class of iterative solvers with the fast property of linear systems of equations with large sparse matrices ascending from PDE or integral equation discretization. The iterative solvers have nice numerical properties, such as low memory demand of O(N). However, these methods have a serious drawback which slows down convergence with the increase of discretization resolution the smoothing property.

Often, iterative solutions reduce high-frequency error components, which are related to the toughest variations of the error spread on the grid or domain. Low frequency (low varying) parts aren't taken out, and the error doesn't go away, but instead becomes smooth.

Moreover, the grid size increases or domain diminution leads to the oscillation of the slowly rotting smooth error, making it accordingly extra vulnerable. Coarsening the grid to accelerate the convergence is very effective, but it spoils the discretization accuracy. From this point, we need to define the grid with diminution of that grid h and the equivalent linear system of equations or linear operator equation as the next equation

(6) $$A^{h}u^{h}=f^{h}.$$

The method for improving the performance of iterative methods, at least at the beginning of the iteration, starts with a good initial iteration. For the typical problem, we can find a good initial iteration by explaining the nearly solution for the problem on the coarse grid and by using a few iterations, which is called smoothing.

**Fig. 1:**
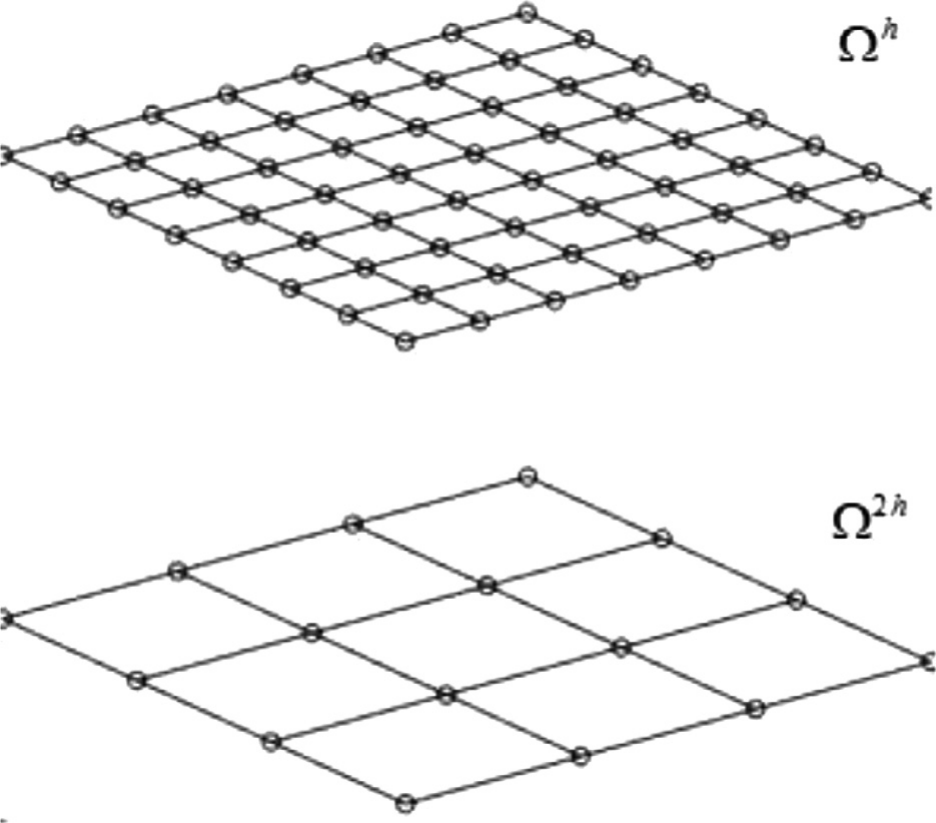
Coarse and fine grid.

Let us study a grid $\Omega^{2h}$. In practice, uniform refinement consists in dividing in halves all intervals of $\Omega^{2h}$, leading to the grid $\Omega^{h}$, see Fig. 1.

*The nested iteration* is the strategy of using coarse grid problems to improve the iterative method to solve (6):

• solve $A^{h}u^{h}=f^{h}$on a very coarse grid approximately by applying a smoother (iteration).
• ⋮
• smooth $A^{2h}u^{2h}=f^{2h}$on $\Omega^{2h}$.
• solve $A^{h}u^{h}=f^{h}$on $\Omega^{h}$ by the iterative method with the initial iteration coming from the coarser grids.

**Fig. 2:**
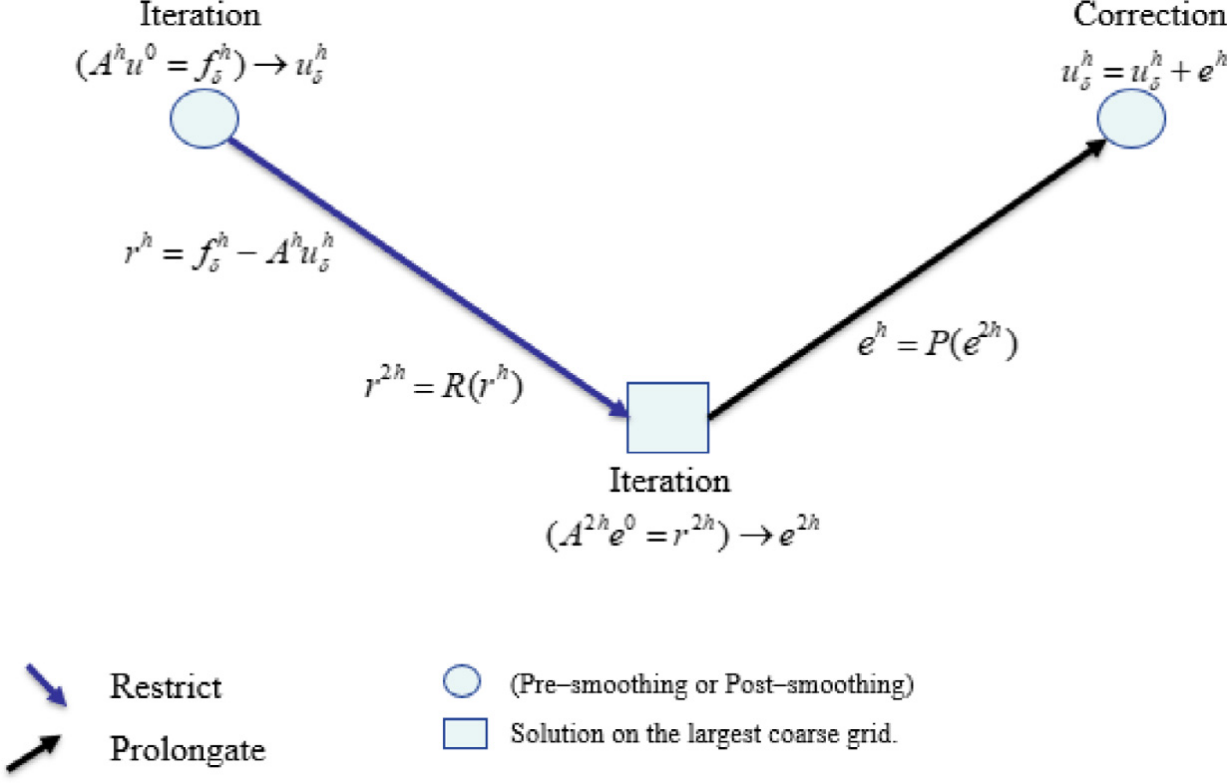
Two-level method.

*Coarse grid correction (two-level method)* is the second strategy and also uses the residual Eq. (5) (see Fig. 2):

• Smooth $A^{h}u^{h}=f^{h}$on $\Omega^{h}$. This step gives an approximation $u_{\delta }^{h}$ of the solution which still has to be updated appropriately. Compute the residual $r^{h}=f_{\delta }^{h}-A^{h}u_{\delta }^{h}$.
• Project by “restrict” residual vector to $\Omega^{2h}$. The result is $R(r^{h})$.
• Solve $A^{2h}e^{2h}=R\left(r^{h}\right)$on $\Omega^{2h}$. With this step, we obtain an approximation of the error, $e^{2h}$.
• Project “prolongate” $e^{2h}$ to $\Omega^{h}$. The result is denoted by $P(e^{2h})$.
• Update the approximation of the solution on $\Omega^{h}$ by $u_{\delta }^{h}=u_{\delta }^{h}+P\left(e^{2h}\right)$

**Fig. 3:**
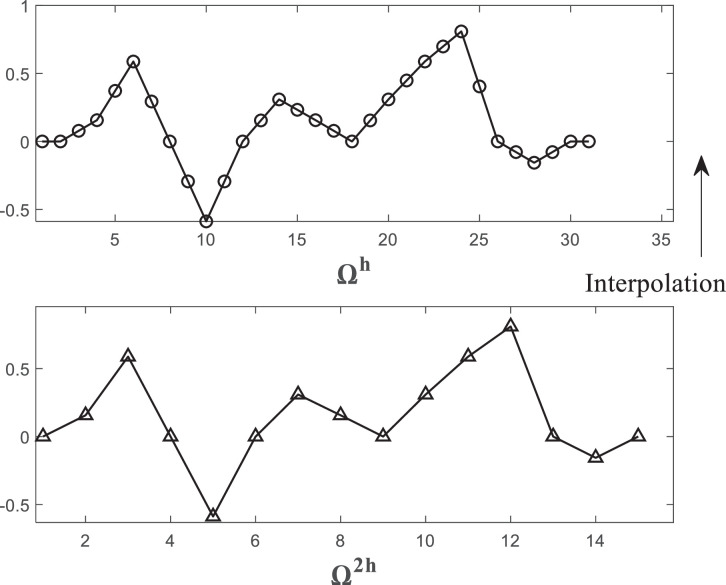
Linear interpolation for finite difference methods.

The transfer from a coarse grid to a fine grid is called prolongation or interpolation. In many situations, we can use simplest approach, which is the linear interpolation. Let $\Omega^{2h}$ be divided into *N* *=* *2* intervals and $\Omega^{h}$ into *N* intervals. The node *j* on $\Omega^{2h}$ corresponds to the node *2j* on $\Omega^{h}$, 0≤j≤N/2, Let $u^{2h}$ be given on $\Omega^{2h}$. Then, the linear interpolation

$$I_{2h}^{h}:R^{N/2-1}\to R^{N-1},u^{h}=I_{2h}^{h}u^{h},$$

is given by

(7) $$\begin{array}{c} u_{2j}^{h}=u_{j}^{2h},j=1,\ldots ,N/2-1,\\ u_{2j+1}^{h}=\frac{1}{2}\left(u_{j}^{2h}+u_{j+1}^{2h}\right),j=0,\ldots ,N/2-1, \end{array}$$

(see Fig. 3). For even points or nodes in grid $\Omega^{h}$, takes the value of the equivalent point in grid $\Omega^{2h}$. About the odd nodes in grid $\Omega^{h}$, the values of the neighbor points is computed.

It can be represented as operator. Using the standard basis of $R^{N/2-1}$ and $R^{N-1}$, then,

(8) $$I_{2h}^{h}u^{2h}=\frac{1}{2}{\left[\begin{array}{ccccc} 1 & & & & \\ 2 & 1 & & & \\ 1 & 2 & & & \\ & 1 & \ddots & & \\ & & \ddots & 1 & \\ & & & 2 & 1\\ & & & 1 & 2\\ & & & & 1 \end{array}\right]}_{\left(n-1\right)\times \left(\frac{n}{2}-1\right)}\left[\begin{array}{c} u_{1}\\ u_{2}\\ \vdots \\ \vdots \\ u_{\frac{n}{2}-1} \end{array}\right]=\left[\begin{array}{c} u_{1}\\ u_{2}\\ u_{3}\\ \vdots \\ \vdots \\ u_{n-1} \end{array}\right]=u^{h}.$$

Suppose that error (unknown) is a smooth vector on the grid $\Omega^{h}$. In addition, the coarse grid estimates on $\Omega^{2h}$ is calculated, and it should be exact in the coarse grid. The interpolation of this coarse grid evaluation is a smooth vector on the fine grid. For this reason, the smooth error on the fine grid has supposed.

**Fig. 4:**
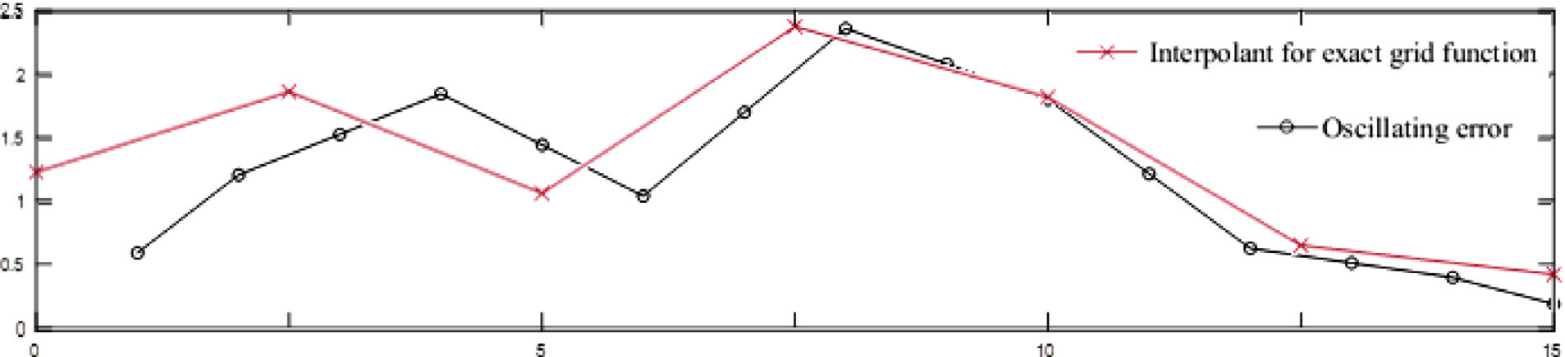
Oscillating error and interpolation.

If there is a fault on the fine grid, then each interpolation of a coarse grid estimate to the fine grid is a smooth function and we cannot expect that the error on the fine grid will come close to being fixed. See Fig. 4.

Perturbation provides good results if the fault on the fine grid is smooth. Hence, prolongation is an appropriate match for the smoother, which works best professionally if the error is wavering.

**Fig. 5:**
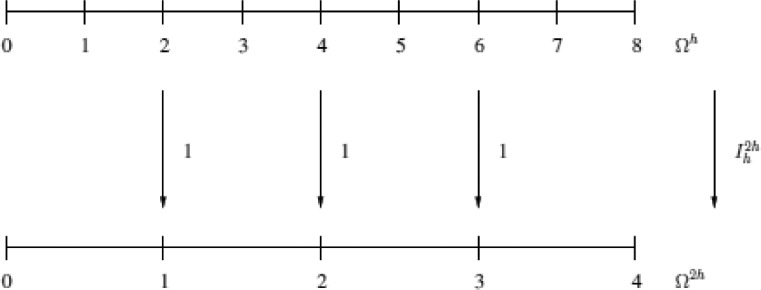
Injection.

For the two-level method, we must do the transmission action for the residual vector from $\Omega^{h}$ to $\Omega^{2h}$ before the coarse grid equation can be solved. This transmission is called restriction. The simplest restriction is injection. It is defined by

$$I_{h}^{2h}:R^{N-1}\to R^{N/2-1},u^{2h}=I_{h}^{2h}u^{h}$$

$$u_{j}^{2h}=u_{2j}^{h},j=1,\ldots ,N/2-1,$$

(see Fig. 5)**.** In this method, for each node on the coarse grid, we take the value of the grid function at the corresponding node on the fine grid.

Injection is an efficient method. If we ignore every other node on h, then the values of the residual in these nodes (and the error in these nodes) have no impact on the system on the coarse grid. Consequently, these errors will generally not be corrected.

There is a weighted restriction for finite difference schemes. The weighted restriction uses all the nodes on the fine grid. It is defined by an appropriate average

(9) $$\begin{array}{c} I_{h}^{2h}:R^{N-1}\to R^{N/2-1},\\ u^{2h}=I_{h}^{2h}u^{h},\;\;u_{j}^{2h}=\frac{1}{4}\left(u_{2j-1}^{h}+2u_{2j}^{h}+u_{2j+1}^{h}\right),j=1,\ldots \frac{N}{2}-1. \end{array}$$

If the spaces $R^{N-1}$ and $R^{N/2-1}$are equipped with the standard bases, the matrix representation of the weighted restriction operator appears as follows:

(10) $$I_{h}^{2h}u^{h}=\frac{1}{4}{\left[\begin{array}{cccccccc} 1 & 2 & 1 & & & & & \\ & 1 & 2 & 1 & & & & \\ & & \ddots & \ddots & \ddots & & & \\ & & & 1 & 2 & 1 & & \\ & & & & & 1 & 2 & 1 \end{array}\right]}_{\left(\frac{n}{2}-1\right)\times \left(n-1\right)}\left[\begin{array}{c} u_{1}\\ u_{2}\\ u_{3}\\ \vdots \\ \vdots \\ u_{n-1} \end{array}\right]=\left[\begin{array}{c} u_{1}\\ u_{2}\\ \vdots \\ \vdots \\ u_{\frac{n}{2}-1} \end{array}\right]=u^{2h}.$$

With this representation, we can see the important connection between weighted restriction $I_{h}^{2h}$and interpolation $I_{2h}^{h}$: $I_{2h}^{h}=2{\left(I_{h}^{2h}\right)}^{T}$

The operator on a coarse grid is defined as the Galerkin projection operation. The operation is starts from the derivation of an appropriate coarse grid operator, by the Galerkin operator which is used by the residual formula as next

(11) $$A^{h}e^{h}=r^{h}.$$

Let us assume that $e^{h}$ is founded in the range of the prolongation operator $I_{2h}^{h}$. Then, there is a vector $e^{2h}$ defined on the next coarse grid as following

(12) $$e^{h}=I_{2h}^{h}\left(e^{2h}\right).$$

Replacing (12) in (11), we obtain

(13) $$A^{h}I_{2h}^{h}\left(e^{2h}\right)=r^{h}.$$

By running the restriction action to both sides of (13) returns

(14) $$I_{h}^{2h}A^{h}I_{2h}^{h}\left(e^{2h}\right)=I_{h}^{2h}r^{h},$$

leading to the definition

(15) $$A^{2h}\left(e^{2h}\right)=r^{2h},$$

where

(16) $$A^{2h}=I_{h}^{2h}A^{h}I_{2h}^{h}.$$

The derivation of (16) was depended on the error *e^h^* is in the range of the prolongation. This property in general is not specified. If it true, then a real solution in coarse grid provide a solution to $A^{h}u^{h}=f^{h}$ with correction step on the coarse grid. However, this derivation gives a motivation for defining $A^{2h}$ as (16).

To make simpler notations, the residual equation for the right-hand side vector will be signified by $f^{2h}$ instead of $r^{2h}$. The solution on the finest grid will represented as $u^{h}$ and the present iterate as $v^{h}$. Instead of representing the solution on the coarse grid as $e^{2h}$, it will be represented as $v^{2h}$.

*Multigrid method V-cycle* is applied by imbedding the two-level method into itself. Let us assume that there some l+1grids, l≥0, where the finest grid has the sizeh and this size increased by the factor 2 for each next or coarser grid. Let $L=2^{l}$.

*Algorithm V-cycle*

• Pre smoothing: apply the smoother $V_{1}$ times to $A^{h}u^{h}=f^{h}$ with the initial guess $v^{h}$. The result is denoted by $v^{h}$.
• Compute $f^{2h}=I_{h}^{2h}\left(f^{h}-A^{h}v^{h}\right).$
  ○ Apply the smoother $V_{1}$ times to $A^{2h}u^{2h}=f^{2h}$ with the initial guess $v^{2h}=0$. Denote the result by $v^{2h}$.
  ○ Compute $f^{4h}=I_{2h}^{4h}\left(f^{2h}-A^{2h}v^{2h}\right).$
  ○ ⋮
  ○ Solve $A^{Lh}u^{Lh}=f^{Lh}$.
  ○ ⋮
  ○ Correct $v^{2h}:=v^{2h}+I_{4h}^{2h}v^{4h}$
  ○ Apply smoother $V_{2}$times to $A^{2h}u^{2h}=f^{2h}$with the initial guess $v^{2h}$.
• Correct $v^{h}:=v^{h}+I_{2h}^{h}v^{2h}$
• Post smoothing: apply smoother $V_{2}$times to $A^{h}u^{h}=f^{h}$with the initial guess $v^{h}$.

**Fig. 6:**
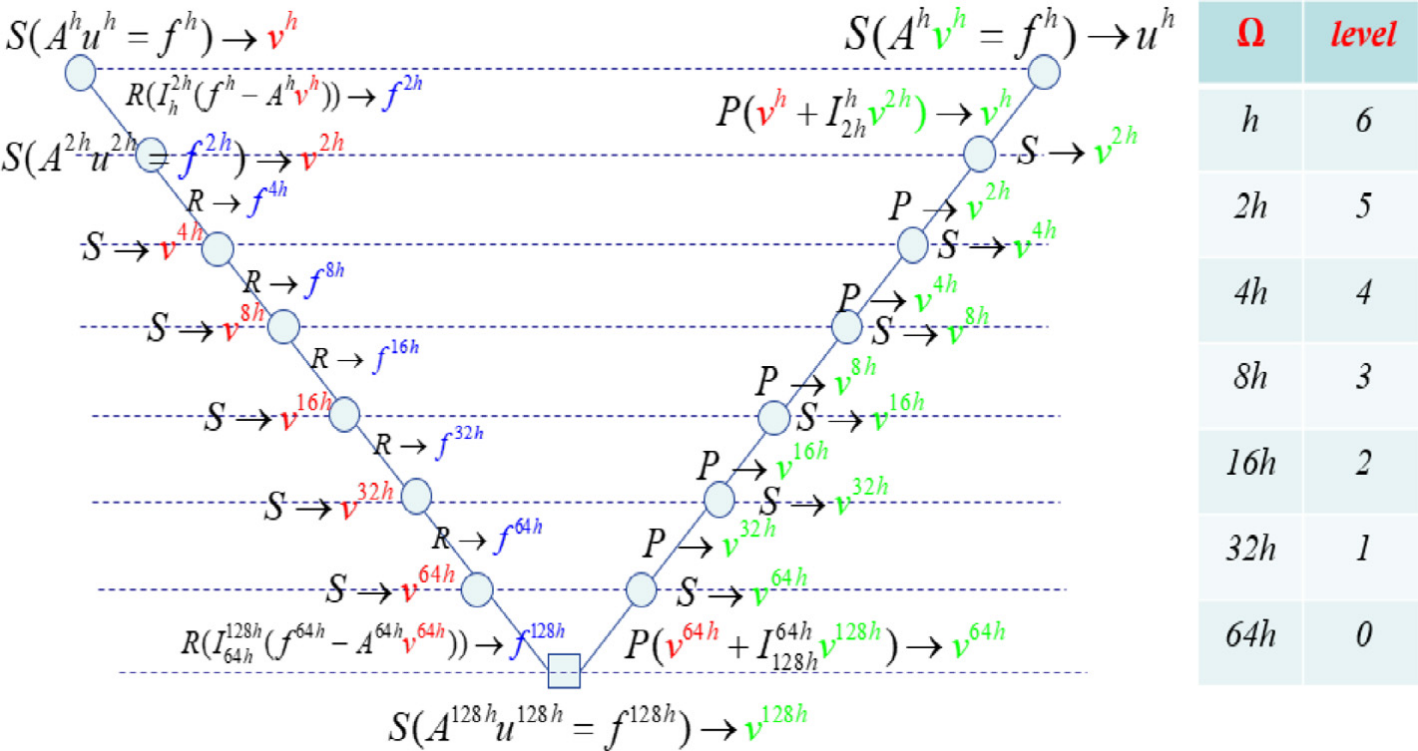
Multigrid V-cycle.

The name *V-cycle* comes from their movement through the hierarchy of grids (see Fig. 6).

**Fig. 7:**
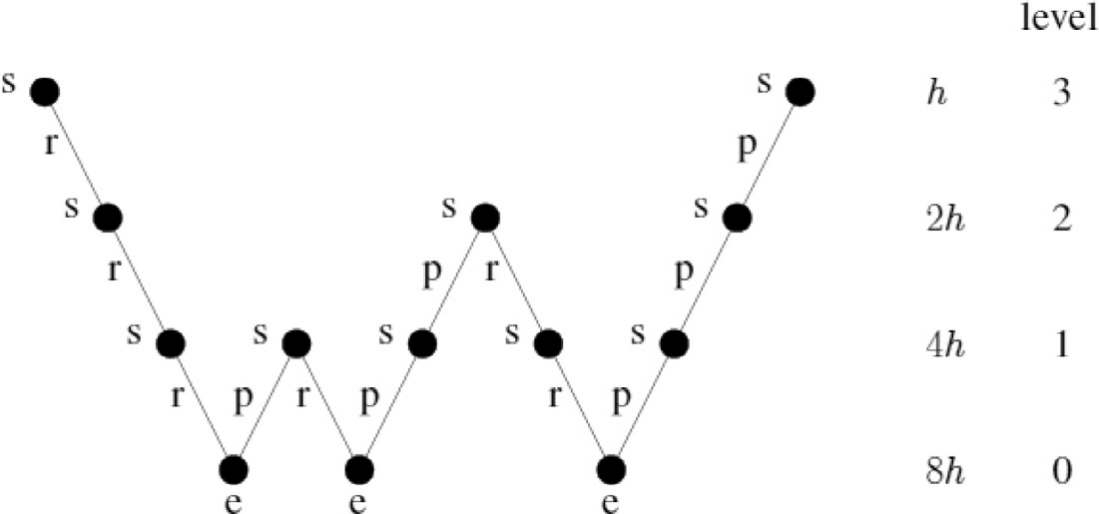
Multigrid W-cycle. *s* smoothing, *r* restriction, *p* prolongated, *e* exact solver.

The v-cycle method is based on the applied recursion of the two*-*level algorithm for solving the coarse grid. The v-cycle algorithm does not provide an answer on how to solve the initial value in the pre-smooth section. There are some other methods, such as the W-cycle (see Fig. 7).

**Fig. 8:**
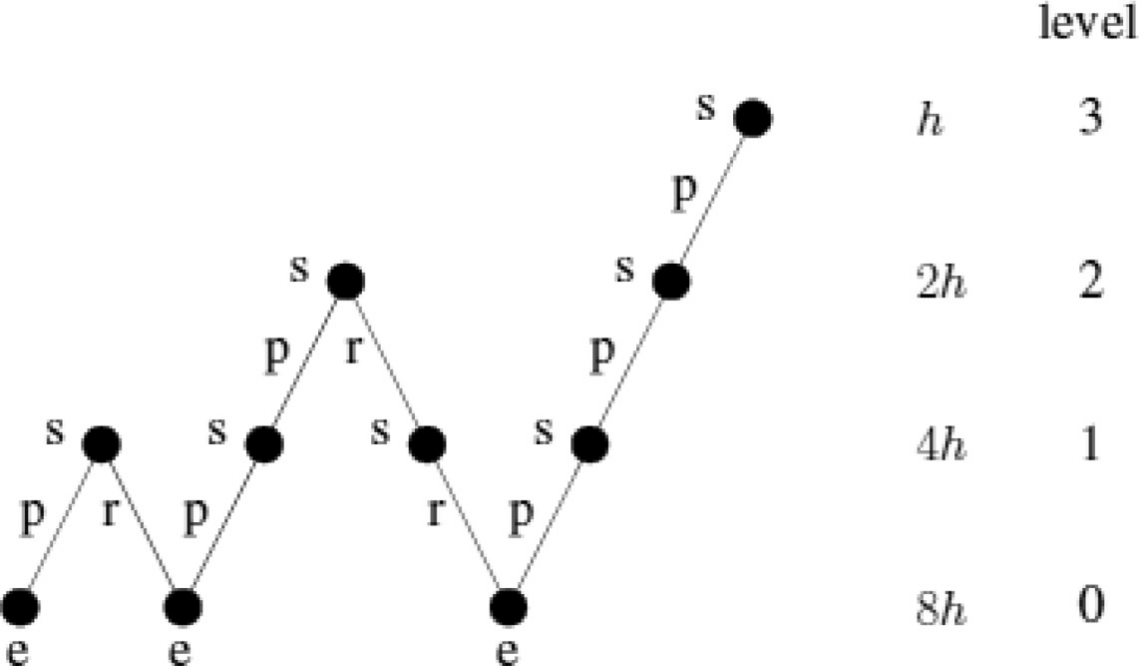
Full multigrid V-cycle.

If we use on each grid (besides the finest grid) one multigrid V-cycle for smoothing, a full multigrid V-cycle is performed (see Fig. 8).

The above methods run sequentially, which requires a great deal of computation time, especially when the fine grid is the highest level. For this reason, we define parallel computing for the V-cycle method as shown in the next section.

## Parallel multigrid method

The two-grid procedure defined above can be comprehensive to an arrangement or next grids through recursion, which leads to the modified FMG with V-cycle as shown in Fig. 6.

**Fig. 9:**
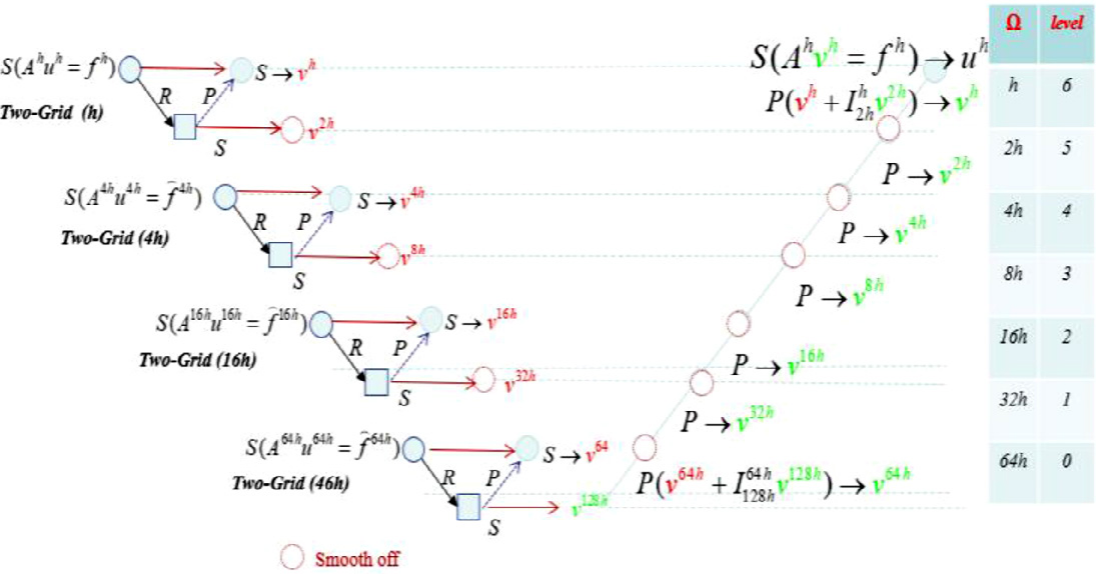
Parallel multigrid V-cycleю *s* smoothing, *r* restriction, *p* prolongation and correcting.

We suggest a parallel method which applies the two-grid method during pre-smoothing for the V-cycle method. In this method, we can compute the residual for each coarse grid independently prior to smoothing. This means that we can apply all pre-smooth computing in parallel as shown in Fig. 9.

The *parallel multigrid method V-cycle* is applied by imbedding the two-level method for each coarse grid prior to smoothing.

*Algorithm PV-cycle*

• Pre smoothing:
  ○ Apply two-grid to $A^{h}u^{h}=f^{h}$, the result is denoted by $v^{h}$ and $v^{2h}$
  ○ Apply two-grid to $A^{4h}u^{4h}=f^{4h}$, the result is denoted by $v^{4h}$ and $v^{8h}$
  ○ ⋮
  ○ Apply two-grid to $A^{(L)h}u^{(L)h}=f^{(L)h}$, the result is denoted by $v^{Lh}$
  ○ ⋮
  ○ Correct $v^{2h}:=v^{2h}+I_{4h}^{2h}v^{4h}$
• Correct $v^{h}:=v^{h}+I_{2h}^{h}v^{2h}$
• Post smoothing: apply smoother $V_{2}$periods to $A^{h}u^{h}=f^{h}$with the initial vector $v^{h}$.

The parallel *V-cycle* becomes a parallel move through the hierarchy of grids, see Fig. 9

To compute the coarse grid operator for each domain independently for each two-grid in the coarse grid, we used the following formula.

(17) $$A^{2^{m}h}=\prod_{j=0}^{m}I_{2^{j}h}^{2^{j+1}h}A^{h}\prod_{j=0}^{m}I_{2^{j+1}h}^{2^{j}h},m=1,2,3,\ldots$$

The same method is used to compute the f right-hand side vector,

(18) $${\overset{⌢}{f}}^{2^{m}h}=\prod_{j=0}^{m}I_{2^{j}h}^{2^{j+1}h}f^{h},m=0,1,2,\ldots$$

**Fig. 10:**
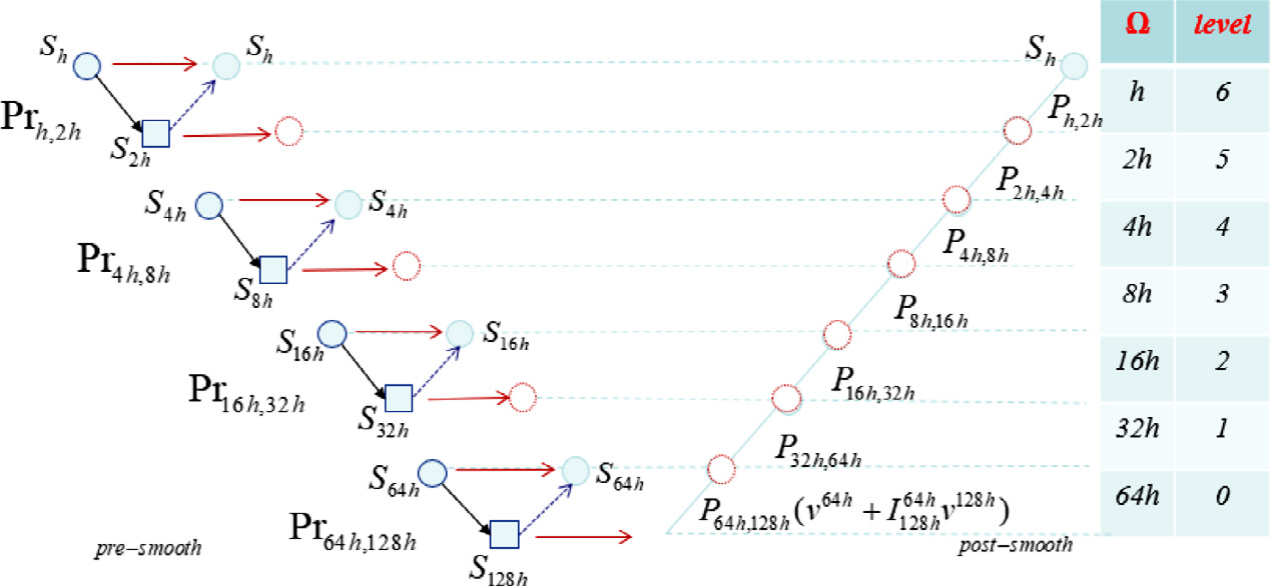
Parallel and sequential processes in V-cycle.

The above-mentioned multigrid algorithms were used in a general-object to solve linear operator equations. In order to compute the computation time for running a parallel V-cycle, we divided the parallel V-cycle into two parts: post-smooth, which is implemented in parallel, and pre-smooth, which is implemented sequentially (see Fig. 10).

**Fig. 11:**
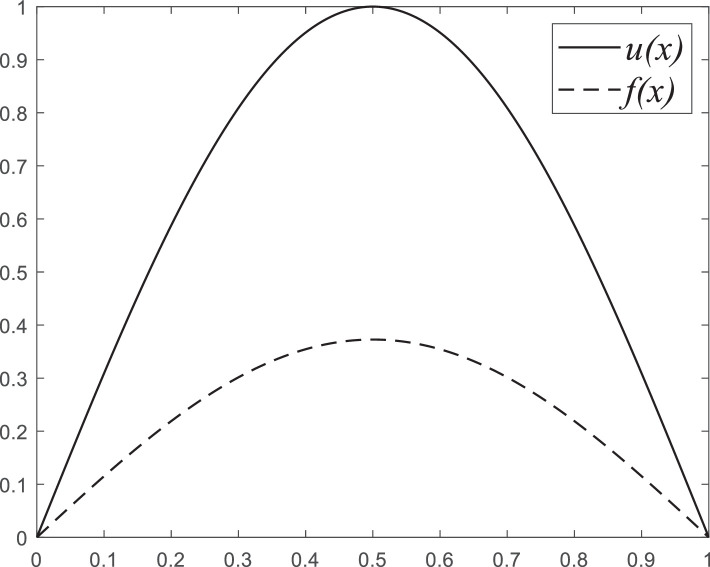
The exact solution for inverse problem and input function.

The time for running each process $Pr_{finegrid,coarsegrid}$ in the pre-smooth ($T_{pre-smooth}$) for each two-grid part is calculated as:

(19) $$T_{pre-smooth}=\max\left\{{\mathrm{Pr}}_{2^{j}h,2^{j+1}h},j=0,1,\ldots m\right\}.$$

We defined the next equation to compute the time for each two-grid process ($S_{h}$smoothing in a fine grid,$S_{2h}$ smoothing in a coarse grid, restriction, and prolongation).

(20) $${\mathrm{Pr}}_{2^{j}h,2^{j+1}h}=2T\left(S_{2^{j}h}\right)+T\left(S_{2^{j+1}h}\right)+T\left(I_{2^{j}h}^{2^{j+1}h}f^{2^{j}h}\right)+T\left(I_{2^{j+1}hh}^{2^{j}h}f^{2^{j+1}}\right),j=0,1,\ldots$$

where $T(S_{2^{j}h})$, returned the time for smoothing, and $T\left(I_{2^{j}h}^{2^{j+1}h}f^{2^{j}h}\right),T\left(I_{2^{j+1}hh}^{2^{j}h}f^{2^{j+1}}\right)$ returned the time for computing the restriction and prolongation operations, respectively.

Regarding post-smoothing computation time $T_{post-smooth}$, the time necessary to complete prolongation with last smooth step can be calculated as:

(21) $$T_{post-smooth}=\sum_{j=0}^{m}T(P_{2^{j}h,2^{j+1}h})+S_{h}.$$

## Numerical experiment and conclusion

The IVP “initial value problem” for heat problem was studied in work [2, 4]. The mathematical form of IVP has been labeled by the following linear PDE system:

(22) $$\frac{\partial u\left(x,t\right)}{\partial t}=\frac{\partial^{2}u\left(x,t\right)}{\partial x^{2}}\;\;\;\;\;0\leq x\;\leq l\;,\;t\in \left(0,T\right],$$

(23) u(0,t)=0,t∈(0,T],

(24) u(l,t)=0,t∈(0,T],

(25) u(x,0)=u(x),0≤x≤l,

where u(0,t) and u(l,t) are boundary conditions and $u_{0}\left(x\right)$ is the IC “initial condition” which is needing to found. In this work, we apply other numerical algorithms to obtain a more accurate solution and fast implementation for a high-scale problem. The integral form for this equation will be:

(26) $$Au\left(x\right)=\int_{0}^{l}\;\;K\left(x,\;y\right)\;u\left(y\right)\;dy=f\left(x\right),$$

where the kernel K(x,y) is an infinite series. Since we cannot hold the infinite sum, we do 10 times for the sum of series:

(27) $$K\left(x,\;y\right)=\frac{2}{l}\sum_{n=1}^{10}\;e^{\frac{-{\left(n\pi \right)}^{2}T}{l^{2}}}\;\text{sin}\left(\frac{n\pi x}{l}\right)\sin\left(\frac{n\pi y}{l}\right),\;\;T>0.$$

To obtain an estimated solution to u(x), we can reformulate the problem as a linear operator equation,Au=f. After applying the discretization algorithm described in [5] we obtain

(28) $$A\left[\begin{array}{c} u\left(y_{0}\right)\\ u\left(y_{2}\right)\\ \vdots \\ u\left(y_{n-1}\right) \end{array}\right]=\left[\begin{array}{c} f\left(x_{0}\right)\\ f\left(x_{2}\right)\\ \vdots \\ f\left(x_{n-1}\right) \end{array}\right],.$$

where

(29) $$A=\frac{1}{n}\left[\begin{array}{cc} \begin{array}{cc} K\left(x_{0},\;y_{0}\right) & K\left(x_{0},\;y_{1}\right)\\ K\left(x_{1},\;y_{0}\right) & K\left(x_{1},\;y_{1}\right) \end{array} & \begin{array}{cc} \ldots & K\left(x_{0},\;y_{n-1}\right)\\ \ldots & K\left(x_{1},\;y_{n-1}\right) \end{array}\\ \begin{array}{cc} \vdots & \vdots \\ K\left(x_{n-1},\;y_{0}\right) & K\left(x_{n-1},\;y_{1}\right) \end{array} & \begin{array}{cc} \ldots & \vdots \\ \ldots & K\left(x_{n-1},\;y_{n-1}\right) \end{array} \end{array}\right].$$

The *bounded and injective operator* Ais ill-conditioned, so any numerical attempt to directly solve (28) will be fail.

Considering the inverse IVP (22)–(25) for the heat equation, we must find $\;u\left(x\right)\in L_{2}\left[0,1\right]$. The exact solution will be u(x)=sinπx,0≤x≤1. We created the input vector or right-hand side for IP “inverse problem” u(x,T)=f(x),0≤x≤1 and T=0.01.

In this numerical example we will use the Landweber method and the same iterative method as in the V-cycle multigrid. We compared the 3 algorithms with high scale of domains $\Omega^{h}$; the result is shown in Table 1 for a domain of $\Omega^{h}=1024$ and Table 2 for domain size $\Omega^{h}=2048$.

**Table 1:** Results for initial value problem $\Omega^{h}=1024$ with δ=0.04.

Algorithm	Process name	parameter			time in second
		No. of iteration	α	The coarsest mesh	
CLI		500	0.1		5.24
V-cycle		15	0.1	2	0.46
P V-cycle I	${\mathrm{Pr}}_{h,2h}$	15	0.1	512	0.18
	${\mathrm{Pr}}_{4h,8h}$	15	0.1	128	0.005
	${\mathrm{Pr}}_{16h,32h}$	15	0.1	32	0.001
	${\mathrm{Pr}}_{64h,128h}$	15	0.1	8	0.0006
	${\mathrm{Pr}}_{256h}$	15	0.1	4	0.0002
	P-post-smooth				0.007

**Table 2:** Results for initial value problem $\Omega^{h}=2048$ with δ=0.05.

Algorithm	Process name	parameter			time in second
		No. of iteration	α	The coarsest mesh	
CLI		500	0.1		20.07
V-cycle		15	0.1	2	1.97
PV-cycle	${\mathrm{Pr}}_{h,2h}$	15	0.1	1024	0.8
	${\mathrm{Pr}}_{4h,8h}$	15	0.1	256	0.02
	${\mathrm{Pr}}_{16h,32h}$	15	0.1	64	0.0016
	${\mathrm{Pr}}_{64h,128h}$	15	0.1	16	0.0005
	${\mathrm{Pr}}_{256h}$	15	0.1	4	0.0003
	P-post-smooth				0.0095

**Fig. 12:**
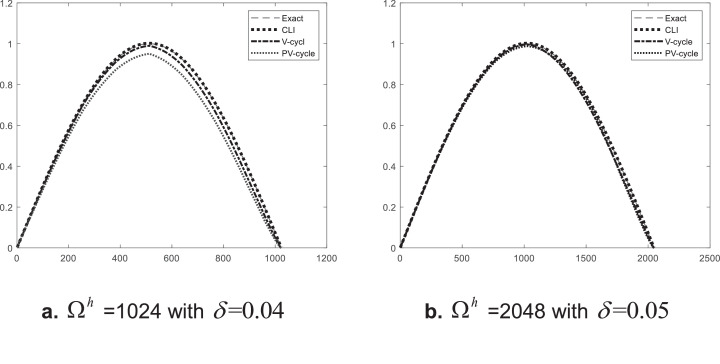
Approximation solutions in $\Omega^{h}$.

The ***V-cycle*** algorithm has been successfully applied to obtain a good approximating solution with low time in high scale domains $\Omega^{h}$, see Fig. 12.

The Landweber iteration method has been successfully applied to obtain an estimated solution. The V-cycle multigrid method has been applied to reduce computation costs for the iterative method. The parallel technique has been successfully applied to the V-cycle multigrid method to obtain the approximation solution for the IVP for a heat equation.

## Conflicts of Interest

The authors declare no conflict of interest.

## Data Availability

No data was used for the research described in the article.
